# Increased Uptake of HCV Testing through a Community-Based Educational Intervention in Difficult-to-Reach People Who Inject Drugs: Results from the ANRS-AERLI Study

**DOI:** 10.1371/journal.pone.0157062

**Published:** 2016-06-13

**Authors:** Perrine Roux, Daniela Rojas Castro, Khadim Ndiaye, Marie Debrus, Camélia Protopopescu, Jean-Marie Le Gall, Aurélie Haas, Marion Mora, Bruno Spire, Marie Suzan-Monti, Patrizia Carrieri

**Affiliations:** 1 INSERM U912 (SESSTIM), Marseille, France; 2 Aix Marseille Université, IRD, UMR-S912, Marseille, France; 3 ORS PACA, Observatoire Régional de la Santé Provence Alpes Côte d'Azur, Marseille, France; 4 AIDES, Pantin, France; 5 Médecins du Monde, Paris, France; University of New South Wales, AUSTRALIA

## Abstract

**Aims:**

The community-based AERLI intervention provided training and education to people who inject drugs (PWID) about HIV and HCV transmission risk reduction, with a focus on drug injecting practices, other injection-related complications, and access to HIV and HCV testing and care. We hypothesized that in such a population where HCV prevalence is very high and where few know their HCV serostatus, AERLI would lead to increased HCV testing.

**Methods:**

The national multisite intervention study ANRS-AERLI consisted in assessing the impact of an injection-centered face-to-face educational session offered in volunteer harm reduction (HR) centers (“with intervention”) compared with standard HR centers (“without intervention”). The study included 271 PWID interviewed on three occasions: enrolment, 6 and 12 months. Participants in the intervention group received at least one face-to-face educational session during the first 6 months.

**Measurements:**

The primary outcome of this analysis was reporting to have been tested for HCV during the previous 6 months. Statistical analyses used a two-step Heckman approach to account for bias arising from the non-randomized clustering design. This approach identified factors associated with HCV testing during the previous 6 months.

**Findings:**

Of the 271 participants, 127 and 144 were enrolled in the control and intervention groups, respectively. Of the latter, 113 received at least one educational session. For the present analysis, we selected 114 and 88 participants eligible for HCV testing in the control and intervention groups, respectively. In the intervention group, 44% of participants reported having being tested for HCV during the previous 6 months at enrolment and 85% at 6 months or 12 months. In the control group, these percentages were 51% at enrolment and 78% at 12 months. Multivariable analyses showed that participants who received at least one educational session during follow-up were more likely to report HCV testing, compared with those who did not receive any intervention (95%[CI] = 4.13[1.03;16.60]).

**Conclusion:**

The educational intervention AERLI had already shown efficiency in reducing HCV at-risk practices and associated cutaneous complications and also seems to have a positive impact in increasing HCV testing in PWID.

## Introduction

Expanded access to needle and syringe programs (NSPs) and opioid maintenance treatment (OMT) have led to a huge decrease in HIV epidemics since the 1990s in France [1] and other countries [2]. However, people who inject drugs (PWID) face other challenges regarding health issues. The Global Burden of Disease project conducted in 77 countries where data on HCV are available showed that HCV prevalence is between 60 and 80% among PWID in 26 countries and >80% in 12 countries, even in countries where harm reduction programs are accessible [3]. Liver disease has become the most common cause of mortality among PWID [4]. The results of modeling of HCV epidemics performed by Vickerman et al. confirmed that scaling-up Opioid maintenance treatment (OMT) and high coverage Needle Syringe programs (NSP) may not be sufficient to reduce HCV prevalence among PWID, and suggested that other interventions are needed [5].

Although PWID are recognised as the population most at risk of HCV infection [3], they are less likely to be referred for HCV testing and care [6]. A Scottish study suggested that only 22% of PWID were diagnosed HCV antibody positive since first attendance at drug centers despite an estimated prevalence of 83% [7]). Other findings showed that 58% of HCV-positive PWID were not HCV-diagnosed [8]. Recent evidence highlighted that PWID who receive positive results for HCV significantly reduce their at-risk practices [9]. Finally, less than 20% of all people who use drugs (PWUD) and who are eligible for HCV treatment are actually treated [10]. In the era of oral direct acting antivirals (DAAs), it seems crucial to increase uptake of HCV testing to improve linkage to care for PWID.

We used data from ANRS-AERLI, a study providing community-based educational training and education about injection, to determine whether the intervention increases uptake of HCV testing among PWID. This secondary analysis evaluated whether this educational intervention increased uptake of HCV testing in this population of difficult-to-reach PWID after adjustment on other known correlates.

## Materials and Methods

### Study design

This national, clustered, multi-site intervention study was conducted in 17 low-threshold drug user harm reduction (HR) centers in France between 2011 and 2013. It enrolled 271 PWID seeking support for their injection practices, including 144 people recruited in 8 HR centers implementing the intervention (hereafter “intervention group”) and 127 people in 9 HR centers not providing the intervention (hereafter “control group”). As explained above, the assignment of HR centers to the intervention and control groups was not randomly performed as it was not feasible to implement the intervention in all HR centers, due to the fact that not all HR centers had a dedicated space and trained staff/volunteers. Therefore, to avoid any possible bias related to non-random assignment, a two-step Heckman model was used in the analysis (see the “Statistical analyses” section). The study included PWID attending the HR center who spontaneously asked for help or information related to injection and who could be reached by phone. Each participant received a small monetary incentive for each questionnaire completed during the phone interview. All PWID who agreed to participate in the study provided written informed consent. The study was approved by the National scientific research ethics committee in Paris. Further details of the study are described elsewhere [11].

### Description of the intervention

The community-based intervention consisted in providing training and education about HIV and HCV transmission risk reduction, with a focus on drug injecting practices, other injection-related complications, and access to HIV and HCV testing and care. It was organized as a series of participant-centered face-to-face educational sessions, taking place in a dedicated room in each intervention group unit. The NGO staff/volunteers followed a comprehensive checklist which covered elements regarding the observation of injection practices and the provision of advice on how to improve them. This was a three-step process:

- Direct observation by trained NGO staff/volunteers of participants’ self-injecting the psychoactive product they habitually used;
- Analysis of each component of the act of injection by the trained NGO staff / volunteers using the checklist to identify associated problems.
- An educational exchange about participant injection practices and questions they might have about a specific element.

Participants had to receive at least one educational session over the first 6 months. The intervention was performed by trained NGO staff or volunteers in the 17 HR centers whose duties include managing NSPs, providing other HR interventions and referring clients for HBV vaccination or HIV and HCV testing.

### Data collection

We used a computer-assisted telephone interview (CATI) to collect the following data at enrolment, M6 and M12: socio-demographic information (gender, age, education level, living in a couple or not, employment status, housing situation), history of drug use (age at first drug injection, type of drugs) and drug and alcohol consumption using, respectively, the Opiate Treatment Index (OTI [12]) and the AUDIT-C questionnaire [13]. With respect to housing, those who reported having their own housing (being owner or renter) were classified as having “stable housing”. We also collected information about access to care, HIV and HCV testing and diagnosis. With respect to HCV testing, patients were asked at enrolment (M0) if they had ever been tested for HCV infection. If they replied yes, subsequent questions collected data about the date of their most recent HCV test and their HCV serostatus (positive, negative, unknown or did not want to disclose). At the two other follow-up visits (M6, M12), participants were asked about HCV testing during the previous 6 months. The status of treatment for opioid dependence was also described. Individuals who reported taking prescribed buprenorphine, methadone or morphine sulfate were considered to be currently on opioid maintenance treatment (OMT). They were also asked whether they engaged in unsafe HIV-HCV transmission practices, using the validated BBV-TRAQ-SV questionnaire [14]. A variable “region” was created by gathering together centers with small population sizes from the same geographic region: Center, North, Paris (one center), and South.

### Study population

**Fig 1:**
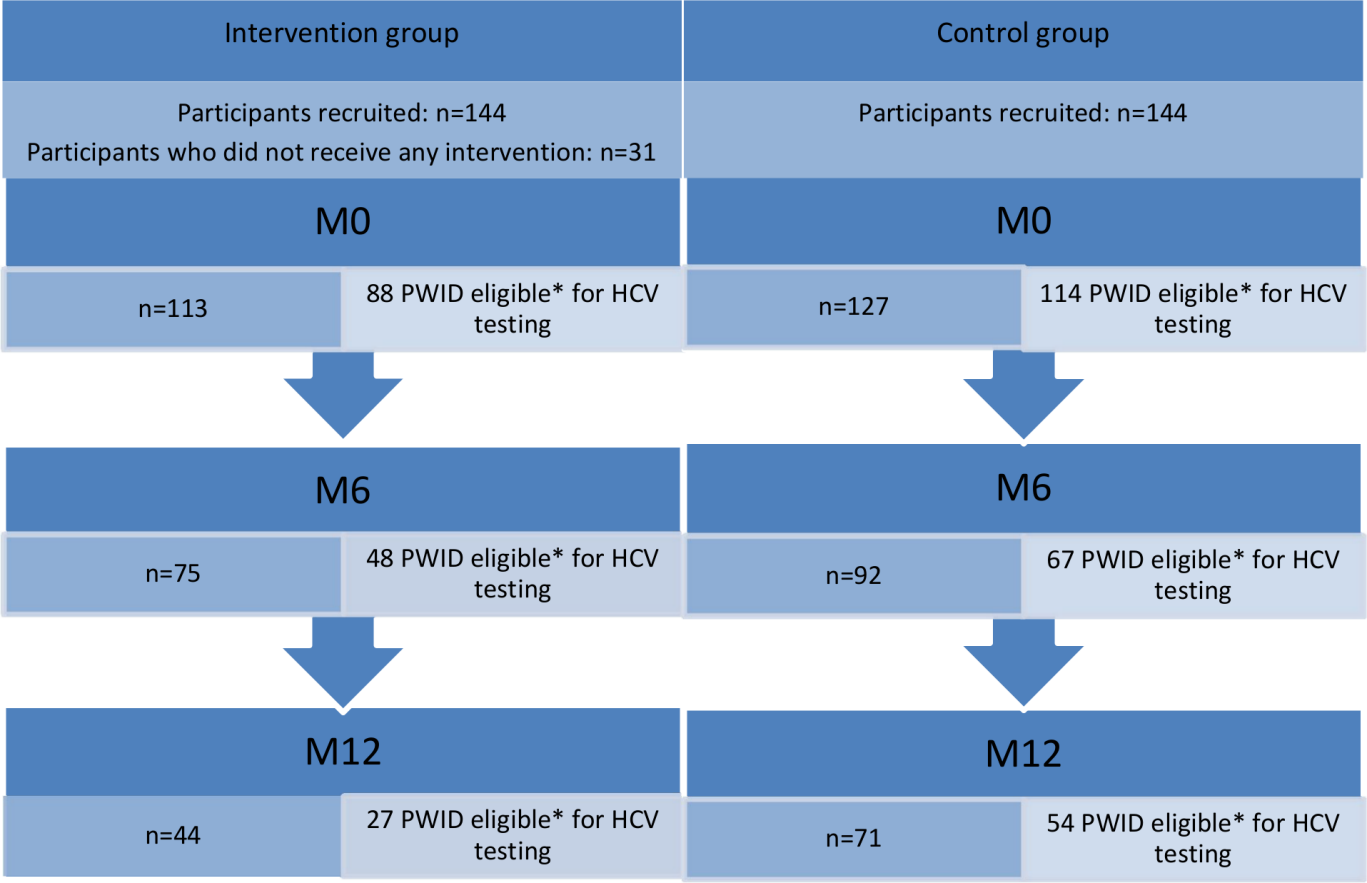
Flow chart–ANRS-AERLI (n = 271).

The study recruited 144 and 127 participants in the intervention and control groups, respectively. Of the former, 31 participants did not receive any educational session during the study and were excluded from analyses. As our outcome was HCV testing uptake at any assessment, of the 240 participants, we only included visits where participants were at risk of HCV infection (i.e. those who were either HCV-negative or whose HCV serostatus was unknown). In addition, we excluded those who reported discordant information regarding their HCV testing and serostatus from one visit to the next (Fig 1). Specific visits (as opposed to participants) were also censored for PWID with a positive HCV serostatus who reported HCV testing during the previous 6 months (for those HCV positive at M0 but with a HCV test during the previous 6 months, we censored M6 and M12 visits; for those HCV positive at M6, we censored M12). The present analysis was performed on a sample of 202 participants (Fig 1).

### Statistical analyses

First, the 31 participants in the intervention group who did not receive any educational session during the study were compared with those who received at least one intervention (n = 113). Furthermore, we compared the 202 participants selected for this analysis with the excluded group (n = 38). In this selected sample, we finally compared participants who received the intervention with those who did not using a Chi-square or exact Fisher test for discrete variables, and a t-Student or Wilcoxon test for continuous variables. In order to take into account the potential bias arising from the non-randomized clustering of groups we initially planned to use a two-step Heckman method adapted for longitudinal settings (details elsewhere [11]) to test the effect of the intervention on the uptake of HCV testing. However, since the inverse Mills ratio (IMR) was not significant in the multivariable Heckman model (p = 0.29), we used a mixed logistic regression model instead. A threshold P-value <0.20 was employed in univariable analyses to identify the variables eligible to enter the multivariable mixed logistic model. A backward procedure was then used to select the explanatory variables for the final multivariable model, with a P-value <0.05. We also tested the interaction effect between follow-up time and receiving the intervention.

## Results

### Baseline characteristics of the sample

First, in the intervention group of the main sample, the 31 participants who did not receive any intervention were not different from those who received at least one intervention during the first 6 months. With respect to the comparison of the 38 participants and the 202 selected, the latter were younger and more likely to report HCV positivity.

The characteristics of the study sample are presented in Table 1. Of the 202 participants analyzed, 23% were female and median age [interquartile range (IQR)] was 30 [25–36] years. One quarter (24%) had a high school certificate, one third (33%) were employed and 27% were living in a couple. One hundred and eleven (55%) had stable housing.

**Table 1:** Baseline characteristics (n (%) or median [IQR]), ANRS-AERLI study (n = 202).

	Control group (n = 114)	Intervention group (n = 88)	P-value	Total (n = 202)
**Gender**			0.99	
male	88 (77)	68 (77)		156 (77)
female	26 (23)	20 (23)		46 (23)
**Age–years** ^§^	30 [26–37]	30 [25–34]	0.36	30 [25–36]
**Education**			0.04	
< High School Certificate	93 (82)	61 (69)		154 (76)
≥ High School Certificate	21 (18)	27 (31)		48 (24)
**Living in a couple**			0.27	
No	87 (76)	61 (69)		148 (73)
Yes	27 (24)	27 (31)		54 (27)
**Employment (paid activity)**			0.21	
No	72 (63)	63 (72)		135 (67)
Yes	42 (37)	25 (28)		67 (33)
**Stable housing**			<0.001	
No	39 (34)	52 (59)		91 (45)
Yes	75 (66)	36 (41)		111 (55)
**Age at first drug injection**^§^	19 [17–23]	20 [17–25]	0.82	20 [17–24]
**Currently on OMT**			0.02	
No	25 (22)	32 (37)		57 (28)
Yes	89 (78)	54 (63)		143 (72)
**Harmful alcohol consumption** ⱡ			0.77	
No	50 (44)	40 (46)		90 (45)
Yes	64 (56)	47 (54)		111 (55)
Heroin use*			0.003	
No	85 (75)	48 (55)		133 (66)
Yes	29 (25)	40 (45)		69 (34)
Cocaine use*			0.47	
No	68 (60)	48 (55)		116 (57)
Yes	46 (40)	40 (45)		86 (43)
Crack use*			0.02	
No	113 (99)	82 (93)		195 (97)
Yes	1 (1)	6 (7)		7 (3)
Buprenorphine use*			<0.001	
No	55 (48)	66 (60)		121 (60)
Yes	59 (52)	22 (40)		81 (40)
Morphine sulfate use*			0.006	
No	80 (70)	45 (51)		125 (62)
Yes	34 (30)	43 (49)		77 (38)
**Frequent daily injection**			0.20	
No	61 (54)	39 (44)		100 (49)
Yes	53 (46)	49 (56)		102 (51)
**HCV screening during lifetime**			0.15	
No	17 (15)	20 (23)		37 (18)
Yes	97 (85)	68 (77)		165 (82)
**Unsafe HIV-HCV transmission practices**^1^			0.05	
No	80 (71)	50 (57)		130 (65)
Yes	33 (29)	37 (43)		70 (35)
**Complications at the injection site**^2^			0.05	
No	52 (46)	28 (32)		80 (40)
Yes	62 (54)	60 (68)		122 (60)
**Self-reported HCV seropositivity**			0.90	
No	97 (86)	69 (85)		166 (86)
Yes	16 (14)	12 (15)		28 (14)
**Self-reported HIV seropositivity**			0.14	
No	105 (96)	81 (100)		186 (98)
Yes	4 (4)	0 (0)		4 (2)

^§^ in years

ⱡ AUDIT-C ≥ 3 for women and ≥4 for men

* during the previous 4 weeks

^1^ at least 1 unsafe HIV-HCV transmission practice during the previous month

^2^ at least 1 complication at the injection site during the previous month.

With respect to drug and alcohol use, median age [IQR] at first drug injection was 20 [17–24] years, while 34% reported heroin use, 43% cocaine use, 3% crack use, 38% morphine sulfate use, and 40% buprenorphine use during the previous month. Fifty-five percent reported harmful alcohol consumption. With respect to treatment status for opioid dependence, we found that 72% of participants at baseline were currently on OMT.

In terms of HCV and HIV, at baseline, 82% reported having been tested for HCV, 35% reported unsafe HIV-HCV transmission practices and 60% had complications at the injection site. Finally, 15 participants self-reported HCV seropositivity, 4 HIV seropositivity. Eight and two, respectively, reported that they did not know their HCV and HIV serostatus.

### Factors associated with being exposed to the intervention

After comparing participants’ baseline characteristics between the intervention and the control groups, we found that those who received at least one educational session during follow-up were more likely to have unstable housing, to have a high school certificate, to have unsafe HIV-HCV transmission practices and complications at the injection site, to use heroin or crack, and not to use buprenorphine or morphine sulfate (Table 1).

### HCV testing during the study

At baseline, 51% of participants in the control group reported HCV testing during the previous 6 months, 34% reported HCV testing more than 6 months previously and 15% no HCV testing. In the intervention group, 44% of participants reported HCV testing during the previous 6 months, 33% reported HCV testing more than 6 months previously and 23% no HCV testing.

**Fig 2:**
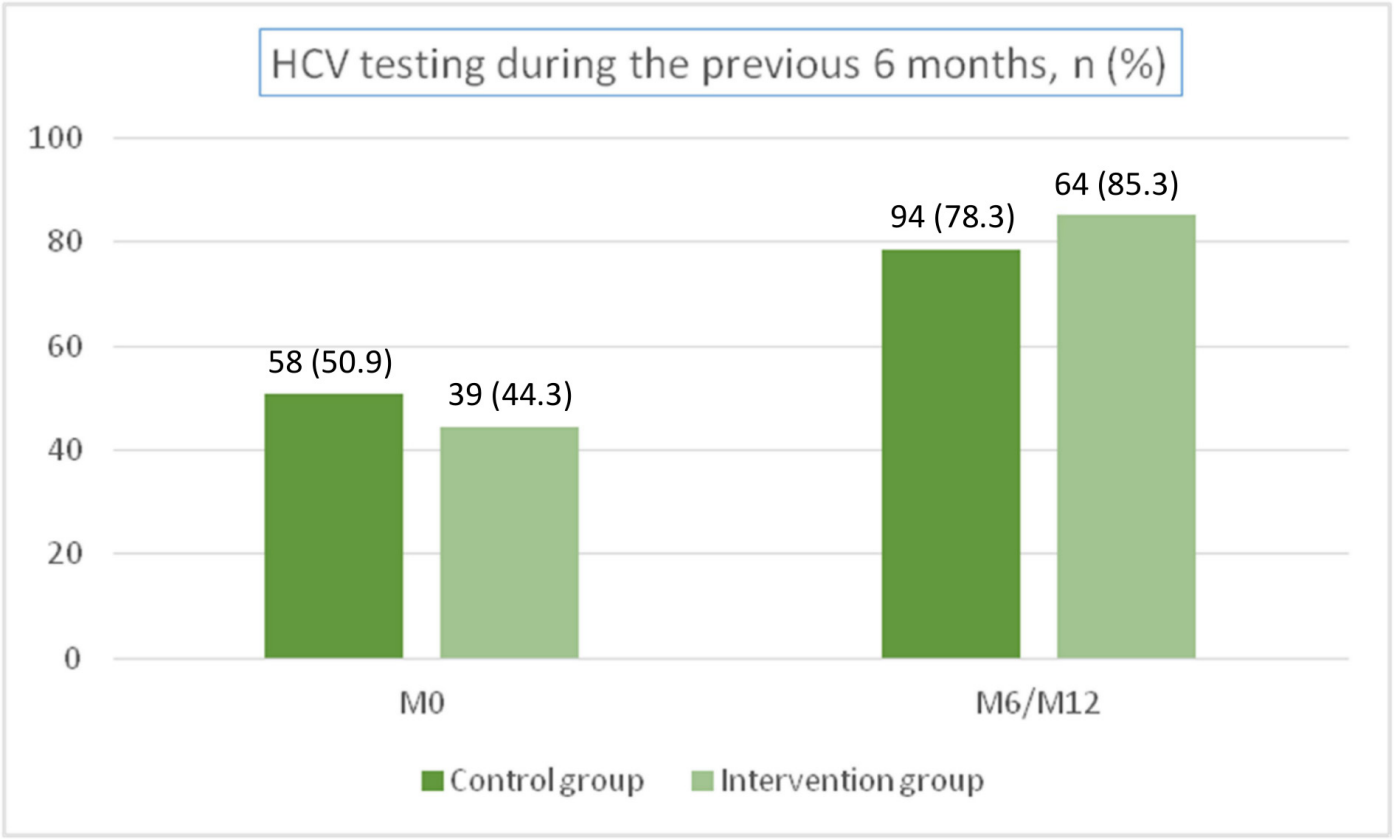
Percentage of participants who reported HCV testing during the previous 6 months; ANRS-AERLI study (n = 202).

At the end of the study, the percentage of participants who reported being tested for HCV during the previous 6 months was 78% in the control group and 85% in the intervention group (Fig 2).

### Impact of intervention on HCV testing

**Fig 3:**
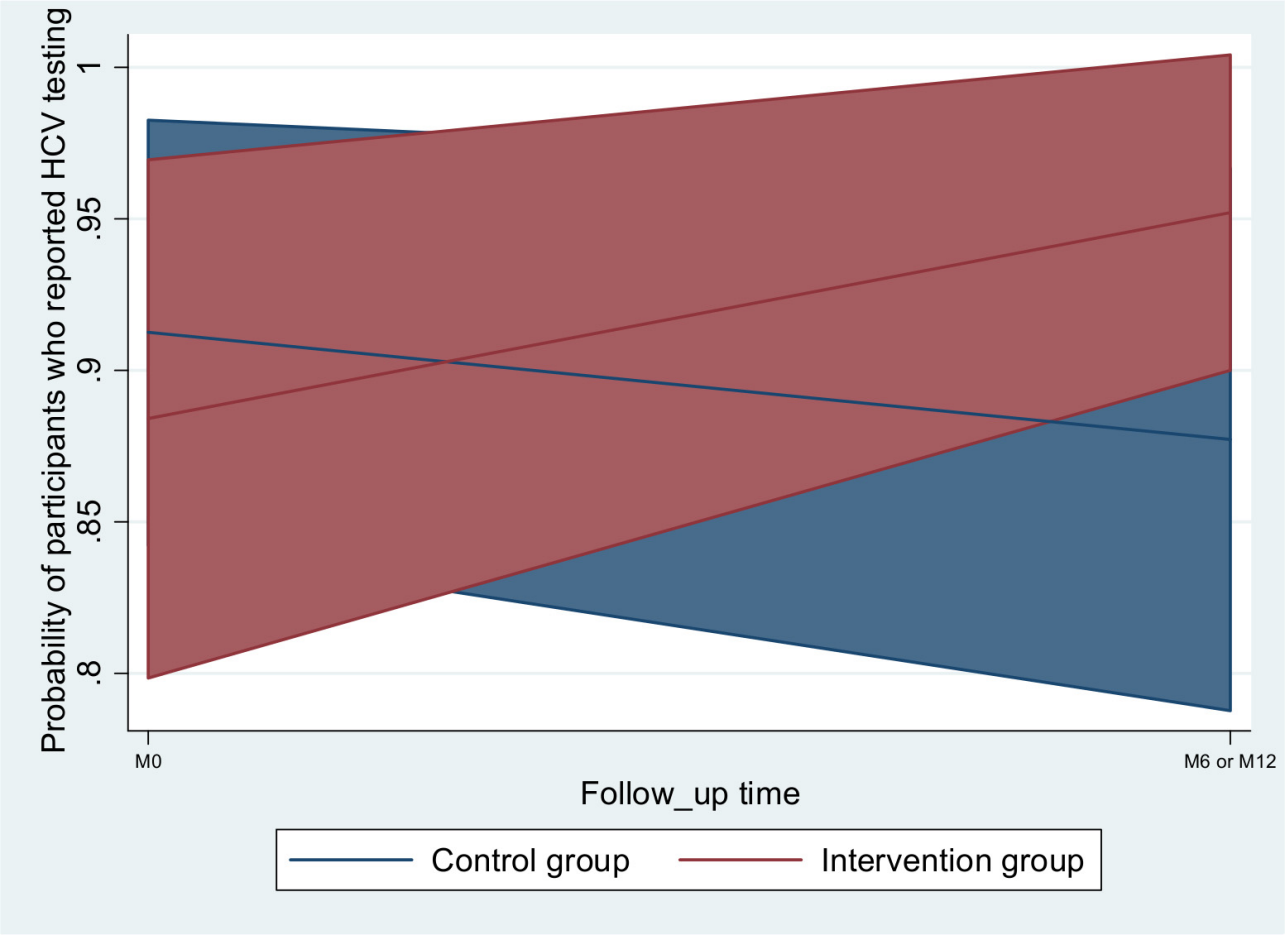
Predictive margins.

The univariable and multivariable analyses, described in Table 2, show the factors associated with HCV testing in the whole study sample (n = 202). After adjusting for two other factors associated with HCV testing (using crack and buprenorphine), we found a significant interaction effect between the intervention group and follow-up (Fig 3), in that participants exposed to the intervention at least once during follow-up were more likely to have been tested for HCV (odds-ratio (OR) [95% confidence interval (CI)] = 4.13 [1.03;16.60] in the intervention group, at M6 or M12 follow-up visits). No significant random regional effect was found.

**Table 2:** Factors associated with HCV testing: logit mixed model, univariable and multivariable analyses, ANRS-AERLI study (n = 202 individuals, N = 395 observations).

	Univariable analyses		Multivariable analysis	
	OR [95%CI]	p-value	aOR [95%CI]	p-value
**Gender**				
male	1			
female	0.55 [0.20; 1.47]	0.23		
**Age**^§^	1.03 [0.97; 1.09]	0.37		
**Education**				
< High School Certificate	1			
≥ High School Certificate	0.84 [0.33; 2.12]	0.71		
**Living in a couple**				
No	1			
Yes	1.37 [0.62; 3.03]	0.44		
**Age at first drug injection**^§^	0.99 [0.92; 1.06]	0.77		
**Stable housing**				
No	1			
Yes	0.58 [0.26; 1.28]	0.18		
**Paid activity**				
No	1			
Yes	1.24 [0.59; 2.60]	0.58		
**Age at first regular drug use**	0.97 [0.91; 1.04]	0.47		
**Currently on OMT**				
No	1			
Yes	1.84 [0.80; 4.22]	0.15		
**Polydrug use**				
No	1			
Yes	0.91 [0.38; 2.17]	0.84		
**Harmful alcohol consumption** ⱡ				
No	1			
Yes	0.72 [0.34; 1.54]	0.40		
Heroin use*				
No	1			
Yes	0.83 [0.39; 1.78]	0.63		
Cocaine use*				
No	1			
Yes	1.48 [0.71; 3.11]	0.30		
Crack use*				
No	1		1	
Yes	0.13 [0.02; 0.84]	0.03	0.11 [0.02; 0.80]	0.030
Morphine sulfate use*				
No	1			
Yes	0.73 [0.34; 1.56]	0.42		
Buprenorphine use*				
No	1		1	
Yes	2.53 [1.07; 5.98]	0.04	2.85 [1.08; 7.53]	0.034
Daily frequent injection**				
No	1			
Yes	1.45 [0.71; 2.95]	0.31		
**Intervention group**				
No	1		1	
Yes	0.85 [0.38; 1.90]	0.69	0.72 [0.24; 2.13]	0.547
**Follow-up time**				
M0	1		1	
M6 or M12	1.13 [0.61; 2.12]	0.69	0.67 [0.28; 1.59]	0.362
**Interaction**				
Intervention group at M0			1	
Intervention group at M6 or M12			4.13 [1.03; 16.60]	0.046

^§^in years

ⱡ AUDIT-C ≥ 3 for women and ≥4 for men

*during the previous 4 weeks

** more than 3 times a day; (a) OR = (adjusted) odds-ratio; CI = confidence interval.

Among all the visits where participants reported buprenorphine injection (n = 150), 90% (n = 135) were on OMT.

## Discussion

This study shows an additional important impact on patient uptake of HCV testing of a community-based educational intervention on injecting practices for PWID conducted in low-threshold sites. Indeed, in this population where HCV prevalence is extremely high and access to care often complicated, our results suggest that this type of intervention may be effective to increase uptake of HCV testing in difficult-to-reach PWID. Recent evidence suggests that receiving positive testing results for HCV significantly reduces at-risk injecting practices among PWIDs [9]. The ANRS-AERLI intervention-based study was designed as an educational support to limit unsafe injecting practices and their consequences in PWID [11]. This is the population most at risk in terms of HCV seropositivity and transmission [15]. They are also much more marginalized from prevention and care for many reasons, including fear of stigma and discrimination [16]. In addition, structural impediments exist, and a large gap between support needs and supply has already been highlighted in homeless individuals with psychiatric issues, especially in the HCV-infected population [17]. Despite outreach interventions, for example mobile units for specific populations [18, 19], HCV testing remains a challenge for PWID, especially for younger injectors who are less likely to be aware of HCV testing and treatment options [20]. This educational intervention could serve as an entry point for prevention and information strategies for HCV screening and care in this marginalized at-risk population.

Besides participation in the AERLI intervention, the two other variables associated with HCV testing are also of interest for our interpretation. The first is that individuals who reported injecting buprenorphine reported being on OMT in over 90% of visits. This reflects the fact that the majority of opioid dependent people on OMT receive buprenorphine through prescription by primary care physicians. In France, OMT coverage is very high with an estimated 138,000 people receiving OMT in 2009, of whom 73% received buprenorphine and 27% received methadone [21, 22]. It has been already shown that access to OMT may increase uptake of HCV testing and care [23]. Although OMT medications, especially buprenorphine (Roux et al., 2008), are sometimes diverted by injection, medical follow-up and current harm reduction interventions which include OMT may have a positive impact on uptake of HCV testing. Even though real world HCV testing practices by primary care physicians in France did not reflect prevailing guidelines at the beginning of the 2000s [24], the prevention campaign implemented in the second half of the 2000s seems to have had a positive impact on increased uptake of HCV testing and care in primary care [25].

The second variable associated with HCV testing was crack use. Participants who reported they were crack users were less likely to have been tested for HCV. This is an important result and highlights the need to provide improved access to prevention and care for this population. It has been already shown that crack users have more limited access to HCV treatment [26, 27]. Furthermore, unlike opioid-dependent individuals who have access to adequate treatment, mainly opioid maintenance treatment, they have no analogous pharmacological treatment. Nevertheless, crack use is increasingly associated with HCV infection and significant social marginalization [28]. Our results show that even with access to this educational intervention, crack users in our study had more limited access to HCV testing. This remains a challenging issue as in France this population are known to be vulnerable to HCV and several studies have found high prevalence in this population [29].

Some limitations of this study should be acknowledged. First, the use of self-reports is known to be subject to social desirability bias. Nevertheless, their reliability in drug-using populations has already been demonstrated [30]. In addition, to limit desirability bias, data were collected using computer-assisted telephone interviews (CATI), conducted by a trained, external, non-judgmental interviewer not involved in the educational sessions. Second, the reliability of using non-randomized clustering to compare the control and intervention groups may be put into question. However, we performed a secondary analysis based on a Heckman model to ensure the two groups could be compared when measuring the effect of the intervention on access to HCV testing. This analysis did not affect the results. Another limitation is that the harm reduction centers who agreed to participate in the study were already more engaged in access to HCV testing and care for drug users. Had the comparison been made with a control group created from centers not already engaged in HCV testing for drug users, it might have been more difficult to see the impact of the intervention between the control and intervention groups. Finally, although we did our best to control for non-randomization bias, the amplitude of the confidence intervals and the risk of uncontrolled differences between the two groups suggest the need to confirm our results in a more controlled setting.

This educational ANRS-AERLI intervention has been shown to be efficient not only in reducing unsafe HCV transmission practices and associated cutaneous complications, but also in increasing HCV testing in PWID. The latter finding may be used in community-based settings to increase the availability of similar educational programs. In addition, further interventions may be adapted to more marginalized populations, especially crack users.

## References

[1] Emmanuelli J, Desenclos JC. Harm reduction interventions, behaviours and associated health outcomes in France, 1996–2003. Addiction 2005,100:1690–1700. 16277629 10.1111/j.1360-0443.2005.01271.x

[2] Degenhardt L, Mathers BM, Wirtz AL, Wolfe D, Kamarulzaman A, Carrieri MP, et al What has been achieved in HIV prevention, treatment and care for people who inject drugs, 2010–2012? A review of the six highest burden countries. Int J Drug Policy 2014,25:53–60. 10.1016/j.drugpo.2013.08.004 24113623

[3] Nelson PK, Mathers BM, Cowie B, Hagan H, Des Jarlais D, Horyniak D, et al Global epidemiology of hepatitis B and hepatitis C in people who inject drugs: results of systematic reviews. Lancet 2011,378:571–583. 10.1016/S0140-6736(11)61097-0 21802134 PMC3285467

[4] Gibson A, Randall D, Degenhardt L. The increasing mortality burden of liver disease among opioid-dependent people: cohort study. Addiction 2011,106:2186–2192. 10.1111/j.1360-0443.2011.03575.x 21749525

[5] Vickerman P, Martin N, Turner K, Hickman M. Can needle and syringe programmes and opiate substitution therapy achieve substantial reductions in hepatitis C virus prevalence? Model projections for different epidemic settings. Addiction 2012,107:1984–1995. 10.1111/j.1360-0443.2012.03932.x 22564041

[6] Wiessing L, Ferri M, Grady B, Kantzanou M, Sperle I, Cullen KJ, et al Hepatitis C virus infection epidemiology among people who inject drugs in Europe: a systematic review of data for scaling up treatment and prevention. PLoS One 2014,9:e103345 10.1371/journal.pone.0103345 25068274 PMC4113410

[7] McDonald SA, Hutchinson SJ, Schnier C, McLeod A, Goldberg DJ. Estimating the number of injecting drug users in Scotland's HCV-diagnosed population using capture-recapture methods. Epidemiol Infect 2014,142:200–207. 10.1017/S0950268813000617 23522183 PMC9152607

[8] McDonald SA, Hutchinson SJ, Mills PR, Bird SM, Robertson C, Dillon JF, et al Diagnosis of hepatitis C virus infection in Scotland's injecting drug user population. Epidemiol Infect 2010,138:393–402. 10.1017/S0950268809990616 19723361

[9] Bruneau J, Zang G, Abrahamowicz M, Jutras-Aswad D, Daniel M, Roy E. Sustained drug use changes after hepatitis C screening and counseling among recently infected persons who inject drugs: a longitudinal study. Clin Infect Dis 2014,58:755–761. 10.1093/cid/cit938 24363333

[10] Grebely J, Genoway KA, Raffa JD, Dhadwal G, Rajan T, Showler G, et al Barriers associated with the treatment of hepatitis C virus infection among illicit drug users. Drug Alcohol Depend 2008,93:141–147. 17997050 10.1016/j.drugalcdep.2007.09.008

[11] Roux P, Le Gall JM, Debrus M, Protopopescu C, Demoulin B, Lions C, et al Innovative community-based educational face-to-face intervention to reduce HIV, HCV and other blood-borne infectious risks in difficult-to-reach people who inject drugs: results from the ANRS-AERLI intervention study. Addiction 2015. 10.1111/add.13089 26234629

[12] Darke S, Hall W, Wodak A, Heather N, Ward J. Development and validation of a multi-dimensional instrument for assessing outcome of treatment among opiate users: the Opiate Treatment Index. Br J Addict 1992,87:733–742. 1591524 10.1111/j.1360-0443.1992.tb02719.x

[13] Bradley KA, DeBenedetti AF, Volk RJ, Williams EC, Frank D, Kivlahan DR. AUDIT-C as a brief screen for alcohol misuse in primary care. Alcohol Clin Exp Res 2007,31:1208–1217. 17451397 10.1111/j.1530-0277.2007.00403.x

[14] Fry CL, Lintzeris N. Psychometric properties of the Blood-borne Virus Transmission Risk Assessment Questionnaire (BBV-TRAQ). Addiction 2003,98:171–178. 12534421 10.1046/j.1360-0443.2003.00207.x

[15] Page K, Morris MD, Hahn JA, Maher L, Prins M. Injection drug use and hepatitis C virus infection in young adult injectors: using evidence to inform comprehensive prevention. Clin Infect Dis 2013,57 Suppl 2:S32–38. 10.1093/cid/cit300 23884063 PMC3722077

[16] Whelan C, Chambers C, Chan M, Thomas S, Ramos G, Hwang SW. Why do homeless people use a mobile health unit in a country with universal health care? J Prim Care Community Health 2010,1:78–82. 10.1177/2150131910372233 23804366

[17] Currie LB, Patterson ML, Moniruzzaman A, McCandless LC, Somers JM. Examining the relationship between health-related need and the receipt of care by participants experiencing homelessness and mental illness. BMC Health Serv Res 2014,14:404 10.1186/1472-6963-14-404 25230990 PMC4179857

[18] Daiski I. The health bus: healthcare for marginalized populations. Policy Polit Nurs Pract 2005,6:30–38. 16443957 10.1177/1527154404272610

[19] Hastings J, Zulman D, Wali S. UCLA mobile clinic project. J Health Care Poor Underserved 2007,18:744–748. 17982203 10.1353/hpu.2007.0097

[20] Vallejo F, Barrio G, Brugal MT, Pulido J, Toro C, Sordo L, et al High hepatitis C virus prevalence and incidence in a community cohort of young heroin injectors in a context of extensive harm reduction programmes. J Epidemiol Community Health 2015,69:599–603. 10.1136/jech-2014-205070 25870164

[21] EMCDDA. Drug treatment overview for France. In: European Monitoring Centre for Drugs and Drug Addiction; 2013.

[22] Benyamina A. The current status of opioid maintenance treatment in France: a survey of physicians, patients, and out-of-treatment opioid users. Int J Gen Med 2014,7:449–457. 10.2147/IJGM.S61014 25228817 PMC4164042

[23] Seidenberg A, Rosemann T, Senn O. Patients receiving opioid maintenance treatment in primary care: successful chronic hepatitis C care in a real world setting. BMC Infect Dis 2013,13:9 10.1186/1471-2334-13-9 23298178 PMC3548742

[24] Rotily M, Loubiere S, Prudhomme J, Portal I, Tran A, Hofliger P, et al [Factors related to screening of hepatitis C virus in general medicine]. Gastroenterol Clin Biol 2002,26:261–269. 11981471

[25] Salmon-Ceron D, Cohen J, Winnock M, Roux P, Sadr FB, Rosenthal E, et al Engaging HIV-HCV co-infected patients in HCV treatment: the roles played by the prescribing physician and patients' beliefs (ANRS CO13 HEPAVIH cohort, France). BMC Health Serv Res 2012,12:59 10.1186/1472-6963-12-59 22409788 PMC3325848

[26] Charlebois A, Lee L, Cooper E, Mason K, Powis J. Factors associated with HCV antiviral treatment uptake among participants of a community-based HCV programme for marginalized patients. J Viral Hepat 2012,19:836–842. 10.1111/j.1365-2893.2012.01648.x 23121361

[27] Alavi M, Raffa JD, Deans GD, Lai C, Krajden M, Dore GJ, et al Continued low uptake of treatment for hepatitis C virus infection in a large community-based cohort of inner city residents. Liver Int 2014,34:1198–1206. 10.1111/liv.12370 24164865

[28] Fischer B, Rehm J, Patra J, Kalousek K, Haydon E, Tyndall M, et al Crack across Canada: Comparing crack users and crack non-users in a Canadian multi-city cohort of illicit opioid users. Addiction 2006,101:1760–1770. 17156175 10.1111/j.1360-0443.2006.01614.x

[29] Jauffret-Roustide M, Le Strat Y, Couturier E, Thierry D, Rondy M, Quaglia M, et al A national cross-sectional study among drug-users in France: epidemiology of HCV and highlight on practical and statistical aspects of the design. BMC Infect Dis 2009,9:113 10.1186/1471-2334-9-113 19607712 PMC2733898

[30] Darke S. Self-report among injecting drug users: a review. Drug Alcohol Depend 1998,51:253–263; discussion 267–258. 9787998 10.1016/s0376-8716(98)00028-3

